# Expressions of miR-30c and let-7a are inversely correlated with HMGA2 expression in squamous cell carcinoma of the vulva

**DOI:** 10.18632/oncotarget.13187

**Published:** 2016-11-07

**Authors:** Antonio Agostini, Marta Brunetti, Ben Davidson, Claes G. Trope, Sverre Heim, Ioannis Panagopoulos, Francesca Micci

**Affiliations:** ^1^ Section for Cancer Cytogenetics, Institute for Cancer Genetics and Informatics, The Norwegian Radium Hospital, Oslo University Hospital, Oslo, Norway; ^2^ Centre for Cancer Biomedicine, University of Oslo, Oslo, Norway; ^3^ Department of Pathology, The Norwegian Radium Hospital, Oslo University Hospital, Oslo, Norway; ^4^ Faculty of Medicine, University of Oslo, Oslo, Norway; ^5^ Department of Gynecology, The Norwegian Radium Hospital, Oslo University Hospital, Oslo, Norway

**Keywords:** HMGA2, miR-30c, let-7a, FHIT

## Abstract

Malignant tumors of the vulva, most of them squamous cell carcinomas, account for only 5% of cancers of the female genital tract. Though little is known about the genetic features of these tumors, the Fragile Histidine Triad (*FHIT*) and High Mobility Group AT-hook 2 (*HMGA2*) genes were found deregulated. We wanted to gain more knowledge about the expression of *HMGA2*-related miRNAs such as miR-30c and let-7a, and whether a correlation exists between the expression of *FHIT* and *HMGA2*, in this tumor type. An inverse correlation was found in-as-much as *HMGA2* was highly expressed (mean fold change 8.8) whereas miR30c and let-7a were both downregulated (mean fold change -3.9 and -2.3, respectively). The consistent overexpression of *HMGA2* found in all tumors adds to the likelihood that this gene is of importance in SCC pathogenesis. Moreover, we came to the conclusion that miRNAs may be the cause of the deregulation of *HMGA2*. Our results also show that SCC of the vulva presents a characteristic molecular pattern with *FHIT* being downregulated whereas *HMGA2* is upregulated.

## INTRODUCTION

Squamous cell carcinomas (SCC) account for 70% of all malignant tumors arising in the vulva. [1]. Little is known about the genetic features of this cancer as only 44 cases of vulvar SCC have been cytogenetically and/or molecularly analyzed [2–6]. Our group has previously reported a chromosome- and array-based comparative genomic hybridization (CGH) analysis of vulvar SCC which showed, among other imbalances, frequent loss of chromosomal band 3p14 [2]. The Fragile Histidine Triad (*FHIT*) tumor suppressor gene, which maps to this band, was found downregulated [2]. Recently, we showed expression of the High mobility group AT-hook 2 gene (*HMGA2*) in 86% (20 out of 23) of vulvar SCC analyzed [7].

An inverse correlation between the expression of *FHIT*, miR-30c, and *HMGA2* was found by Suh et al. [6] in lung cancer. *FHIT* enhances the expression of miR-30c as well as *HMGA2* repression thereby inhibiting epithelial-mesenchymal transition and the metastatic process in small cell carcinoma of the lung. The interaction between this miRNA and the *HMGA2* 3’untranslated region (3’UTR) seems to be crucial in gene expression control [8]. Since our previous findings in SCC of the vulva [2] hint at a similar expression correlation as that detected in lung cancer, we decided to quantify the expression levels of *HMGA2* and miR-30c in the 10 tumors of the vulva that were found to have deregulated *HMGA2* and *FHIT*, and to assess the expression levels of the miRNA let-7a which is known to target and repress *HMGA2* [9–11], as well as the expression of the let-7a regulators *LIN28A* and *LIN28B* [12].

## RESULTS

**Figure 1:**
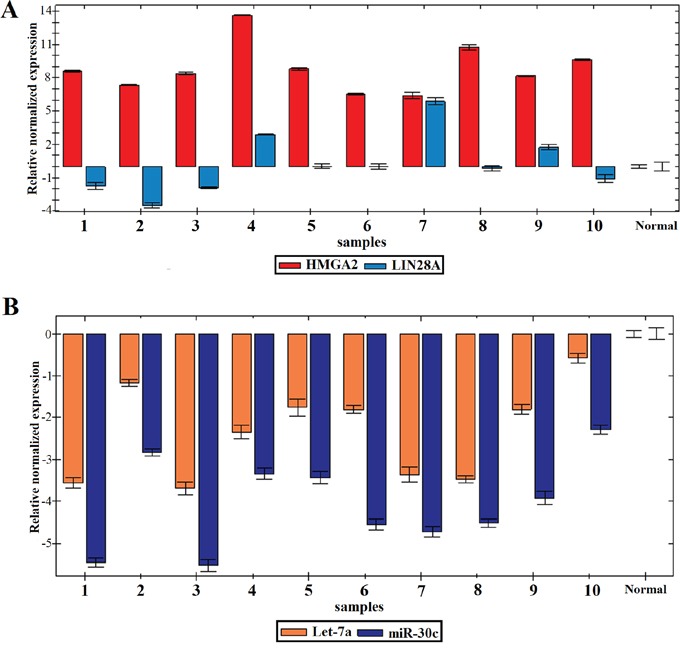
Expression profile of the 10 SCC of the vulva **A.** Relative normalized expression of *HMGA2* and *LIN28A.* **B.** Relative normalized expression of miR-30c and let-7a.

All molecular investigations gave informative results (Table 1) which were normalized using the two normal vulva tissue samples. *HMGA2* was expressed at high levels in all 10 tumors analyzed with an 8.8 fold change average (range: 7-14) (Figure 1A). miR-30c was strongly downregulated in all 10 tumors (Figure 1B) with an average fold change of -3.9 (range: -2.3 to -5.5). The analysis of let-7a expression found downregulation in all cases with a mean -2.3 fold change (range: -0.5 to -3.5) (Figure 1). *LIN28A* was found deregulated in four out of ten tumors. In these samples, the detected fold changes were -1.5 for cases 1 and 2, - 3.5 for case 3, and -1 for case 10 with a mean fold change of -1.8. Three tumors (cases 4, 7, and 9) showed upregulation of *LIN28A* with a fold change of 2.5 for case 4, 6 for case 7, and 1.5 for case 9 giving a mean fold change of 3.3. In the remaining three tumors (cases 5, 6, and 8), *LIN28A* expression was similar to that of the normal controls (Figure 1A). No expression of *LIN28B* was detected in the 10 SCC of the vulva or in the two normal controls. However, the gene was found expressed in the Total Human Universal Reference used as an internal control for each reaction.

**Table 1:** Overview of the samples and results

Case/Lab Number	Diagnosis^a^	FHIT	miR-30c	LIN28A	LIN28B^d^	Let-7a	HMGA2
1/02-167	SCC m	**↓**	**↓**	**↓**	-	**↓**	↑
2/02-848	SCC m	**↓**	**↓**	**↓**	-	**↓**	↑
3/02-869	SCC h, m, p	**↓**	**↓**	**↓**	-	**↓**	↑
4/02-1171	SCC	**↓**	**↓**	↑	-	**↓**	↑
5/03-830	SCC m	**↓**	**↓**	ne^c^	-	**↓**	↑
6/03-1011	SCC h, m	↑	**↓**	ne^c^	-	**↓**	↑
7/03-1088	SCC h	**↓** loss 3p14^b^*	**↓**	↑	-	**↓**	↑
8/06-19	SCC p	**↓**	**↓**	ne^c^	-	**↓**	↑
9/06-125	SCC m	**↓** loss 3p14^b^	**↓**	↑	-	**↓**	↑
10/09-733	SCC h	**↓** loss 3p14^b^	**↓**	**↓**	-	**↓**	↑

Arrows are used to show deregulation of the genes (arrow head up: up regulation; arrow head down: down regulation)

a h: highly differentiated, m: moderately differentiated, p: poorly differentiated.

b Genomic Imbalances assessed with an array based Comparative Genomic Hybridization (aCGH) showing a loss of the *FHIT* locus.

* aCGH detected a homozigous deletion for the *FHIT* locus.

c ne: normal expression (no differences between samples and the normal controls expression levels).

d No *LIN28B* expression was found in our samples.

Searching for possible causes behind the deregulation of let-7a and miR-30c in vulvar SCC, we checked the genomic imbalance charts of these tumors to find if the miRNA clusters for let-7a and miR-30c were deleted. These data were retrived from our previous publication on the very same samples in which an array Comparative Genomic Hybridization study was described [2], looking specifically for the three genes expressing let-7a, *MIRLET7A1* on 9q22.3, *MIRLET7A2* on 11q24.1, and *MIRLET7A3* on 22q13.3, and the two genes for miR-30c, *MIR30C1* and *MIR30C2*, mapping on 1p34.2 and 6q13, respectively. No genomic loss was found for the chromosomal bands in which these miRNAs clusters are located, the only exception being case 10, where a deletion was found in the chromosome arms containing all three *MIRLET7A* genes (6q, 9q, and 22q).

## DISCUSSION

Few SCC of the vulva have been characterized both at the cytogenetic and molecular level. *HMGA2* is one of the few genes found to be activated in this tumor type [6]; however, no previous study has quantified its expression. We found that *HMGA2* was expressed at high levels in all 10 vulvar SCC analyzed with an average fold change of 8.8. HMGA2 belongs to the High-mobility group AT-hook family of non-histone proteins involved in a wide variety of cellular processes from DNA damage repair to gene regulation [13]. The gene is expressed during embryonic development [14] but is usually unexpressed in adult normal tissues [15]. High expression levels of *HMGA2* have been found in various types of tumors including benign connective tissue neoplasms and malignant tumors [16]. HMGA2 promotes diverse tumorigenic processes from cellular proliferation to epithelial-mesenchymal transition (EMT) and metastasis formation [17]. The mechanisms that lead to unchecked expression of *HMGA2* in cancer are still not fully understood, but miRNA-dependent regulation seems to play a role. The *HMGA2* 3’untranslated region (3’UTR) contains regulatory sequences that are targeted by different families of miRNAs [8], and miRNA-dependent repression seems to be the main mechanism to ensure negative *HMGA2* expression [10, 11, 18]. Another piece of evidence testifying to the importance of the interaction between *HMGA2* and miRNAs is the high frequency of disrupted forms of this gene, due to rearrangement of chromosomal band 12q15, found in different tumors [19]. These alterations bring about loss of the 3’UTR leading to a truncated transcript that evades miRNA-dependent gene silencing [19]. Interestingly, no truncated form of the gene was found in the investigated samples [6], further hinting at the importance of alternative mechanisms of gene deregulation in vulvar SCC. To test this further, we decided to assess the expression of two miRNAs, miR-30c and let-7a, that are known to target and regulate *HMGA2* [9, 18].

Downregulation of miR-30c was found in all cases analyzed. For the first time in SCC of the vulva, we showed an inverse correlation betrween miR-30c and *HMGA2* expression similar to the one previously reported in lung cancer [18]. Moreover, when retrieving data on *FHIT* expression from our previous publication [2], we saw similar downregulation of *FHIT* and miR-30c, admittedly with one sample (case 6) showing a high expression of *FHIT* in spite of miR-30c downregulation. This indicates that even other causes of miR-30c downregulation may exist in vulvar SCC.

We found downregulation of let-7a in all tumors analyzed. Deregulation of the let-7 family of miRNAs occurs in different types of tumors and the most important result of it seems to be *HMGA2* overexpression [20, 21]. This is also the case for SCC of the vulva, where deregulation of *HMGA2* could be brought on by altered expression of *FHIT*, miR-30c, and/or let-7a. Allegedly, deregulation of the let-7 family of miRNA is caused by overexpression of the RNA-binding protein homologues LIN28A and LIN28B that inhibit the maturation of both pri-let-7 and pre-let-7 [12]. Since only three out of ten tumors had overexpression of *LIN28A*, we conclude that LIN28A is not the primary cause of let-7a downregulation in SCC of the vulva. The correlation between let-7a downregulation and *HMGA2* overexpression was previously found also in SCC of the oral cavity [22] and esophagus [23]. These findings strengthen our interpretation and lead us to assume that miRNA deregulation is the major cause of the high *HMGA2* expression levels in SCC of the vulva, possibly in squamous cell carcinomas generally.

Our study showed that SCC of the vulva presents a characteristic molecular signature in that *FHIT* is downregulated while *HMGA2* is upregulated. The deregulation of these genes may be correlated with the invasive capacity of these tumors as shown in lung cancer [18]. *FHIT* is a suppressor gene with a role in apoptosis and epithelial-mesenchymal transition (EMT) [24, 25]. HMGA2 induces EMT and invasiveness in epithelial tumors and high expression levels of the gene have been seen in primary as well as metastatic carcinomas [26]. HMGA2 enhances the TGFβ-SMADs signaling pathway [27] and so upregulates the major transcription factors involved in EMT, i.e, SNAIL1, SNAIL2, and TWIST. The consistent overexpression of HMGA2 found in 10 out of 10 tumors adds to the likelihood that this gene is of general importance in SCC pathogenesis. MiRNAs may be the cause of the HMGA2 deregulation. This irrespective, the deregulation of these genes together with downregulation of *FHIT* constitutes a molecular signature of vulvar SCC.

## MATERIALS AND METHODS

### Tumor material

The material consisted of fresh frozen samples from 10 SCC of the vulva and two normal vulva specimens surgically removed at The Norwegian Radium Hospital (Table 1). The tumors have previously been examined for chromosomal aberrations and genomic imbalances as well as for expression of *FHIT* [2, 4] and *HMGA2* [6]. The study was approved by the regional ethics committee (Regional komité for medisinsk forskningsetikk Sør-Øst, Norge, http://helseforskning.etikkom.no) and written informed consent was obtained from the patients.

### Total RNA extraction

Total RNA was extracted using the miRNeasy Kit (Qiagen, Hilden, Germany) and QIAcube (Qiagen) according to the manufacturers’ recommendations. RNA concentration and purity was measured using a Nanovue Spectrophotometer (GE Healthcare, Pittsburgh, PA, USA).

### Real-time polymerase chain reaction (real-time PCR)

The expression of the target miRNAs and genes was assessed with Real-Time PCR. The PCR analyses were performed using the CFX96 Touch Real-Time PCR detection system (Bio-Rad Laboratories, Oslo, Norway). The reactions were carried out in quadruplicate using the TaqMan Universal Master Mix II with UNG (Applied Biosystems, Foster City, CA, USA) following the manufacturer's protocol. Human Universal Reference Total RNA (Clontech, Mountain View, CA, USA) was used as internal reaction control with two samples of normal vulva tissue being used for normalization.

### MicroRNA expression

Ten ng of total RNA were reverse transcribed with the TaqMan MicroRNA Reverse Transcription Kit (Applied Biosystems) following the manufacturer's protocol. miRNA expression was assessed with Real-Time PCR using the TaqMan MicroRNA Assays (Applied Biosystems) for let-7a (TM:000377) and miR-30c (TM:000419). The *RNU6B* gene (TM:001093) was used as a reference.

### Gene expression

One μg of extracted total RNA was reverse-transcribed in a 20 μl reaction volume using iScript Advanced cDNA Synthesis Kit according to the manufacturer's instructions (Bio-Rad Laboratories, Oslo, Norway). Gene expression was assessed with Real-Time PCR using the TaqMan Gene Expression Assays (Applied Biosystems): *HMGA2* (Hs_04397751_m1), *FHIT* (Hs_00179987_m1), *LIN28A* (Hs_00702808_s1), *LIN28B* (Hs_01013729_m1), and *ACTB* (Hs_01939407_gH). The latter gene was used as a reference.
